# Electromagnetic Sensors for Underwater Scour Monitoring

**DOI:** 10.3390/s20154096

**Published:** 2020-07-23

**Authors:** Andrea Maroni, Enrico Tubaldi, Neil Ferguson, Alessandro Tarantino, Hazel McDonald, Daniele Zonta

**Affiliations:** 1 Department of Civil and Environmental Engineering, University of Strathclyde, Glasgow G1 1XQ, UK; enrico.tubaldi@strath.ac.uk (E.T.); n.s.ferguson@strath.ac.uk (N.F.); alessandro.tarantino@strath.ac.uk (A.T.); daniele.zonta@strath.ac.uk (D.Z.); 2 Transport Scotland, Glasgow G4 0HF, UK; hazel.mcdonald@transport.gov.scot

**Keywords:** Bridge scour, scour inspection, structural health monitoring, electromagnetic sensor

## Abstract

Scour jeopardises the safety of many civil engineering structures with foundations in riverbeds and it is the leading cause for the collapse of bridges worldwide. Current approaches for bridge scour risk management rely mainly on visual inspections, which provide unreliable estimates of scour and of its effects, also considering the difficulties in visually monitoring the riverbed erosion around submerged foundations. Thus, there is a need to introduce systems capable of continuously monitoring the evolution of scour at bridge foundations, even during extreme flood events. This paper illustrates the development and deployment of a scour monitoring system consisting of smart probes equipped with electromagnetic sensors. This is the first application of this type of sensing probes to a real case-study for continuous scour monitoring. Designed to observe changes in the permittivity of the medium around bridge foundations, the sensors allow for detection of scour depths and the assessment of whether the scour hole has been refilled. The monitoring system was installed on the A76 200 Bridge in New Cumnock (S-W Scotland) and has provided a continuous recording of the scour for nearly two years. The scour data registered after a peak flood event (validated against actual measurements of scour during a bridge inspection) show the potential of the technology in providing continuous scour measures, even during extreme flood events, thus avoiding the deployment of divers for underwater examination.

## 1. Bridge Scour

Scour is a soil-structure interaction phenomenon that is defined as the erosion of riverbed material surrounding foundations of structures immersed in water [1]. This phenomenon poses a significant risk to bridges crossing rivers and channels, reducing the load-bearing capacity of foundations and causing the bridge to fail and collapse, often without any warning [2]. Thus, monitoring and detecting scour at early stages of development is of paramount importance to ensure the operability and safety of bridges.

Scour initiates when the shear force at the water-bed interface is higher than the critical shear stresses corresponding to the initiation of motion of the soil particles [3]. The type of bed material also plays an essential role in the scour process as the critical shear stress is peculiar to it [4].

Three types of scour generally occur: degradation scour [5], constriction (or contraction) scour [6,7] and local scour [1]. In general, the total scour is the resultant of these three types of scours working simultaneously [6].

Flood-induced scour is recognised as one of the most common causes of bridge failures worldwide. At least 138 railway bridge failures occurred due to scour between 1846 and 2013 in the UK, which in terms of failure rate means one bridge every 2.44 years [8]. In contrast, in the United States, it has been estimated that an average annual rate of 22 bridges collapse or are closed due to scour [9]. Moreover, a review of bridge collapses in the US in the 1990s carried out by Wardhana and Hadipriono [10] shows that the combined figure of 266 flood/scour-related cases constitutes the most dominant bridge failure cause (53% of the total cases of failures).

Current practice for assessing the scour risk of bridges relies on visual inspections at regular intervals, which can involve the use of scuba divers. During an extreme weather event, transport operators make the decision to close the bridge to traffic by visually comparing the water level with a fixed flood level marker [11,12] as it is not possible to carry out underwater inspections due to safety issues. Visual inspections are generally expensive, time-consuming, and the outcomes are often subjective, depending on the inspector’s experience. Furthermore, using the water level only to trigger decisions ensures that the bridge is not inundated or possibly struck with floating debris while open to traffic, but it does not allow for the direct control of scour risk under floods with return periods, other than the one considered for defining the fixed flood level marker.

This monitoring approach has many other limitations. For instance, a very intense flood with a high-flow rate (thus corresponding to a high-water level) does not necessarily correspond to the development of a significant scour hole at the pier if the duration of the flood event is short. Furthermore, measurements of scour carried out after the heavy flood may not capture the maximum scour that occurred during the event, as the scour hole might have partly filled during the recession (i.e., live-bed scour regime) [13]. At the same time, the safety of a bridge could be jeopardised by the progressive accumulation of the excavations under multiple events with low return period (i.e., corresponding to water levels below the marker) occurring in sequence, as was the case of the Lamington viaduct in South-West Scotland [14].

In summary, visual inspections and the water level are very rough indicators of the scour risk. One way to overcome this issue is to equip the structure with Structural Health Monitoring (SHM) systems, providing quantitative information about the extent of scour at bridge foundations and/or the state of the structure condition. Although many SHM systems have emerged in recent years for monitoring bridge scour, very few have been implemented in real practice.

This paper describes the concept, installation and functioning of a pilot scour monitoring system based on the use of smart electromagnetic probes and deployed on the A76 200 bridge over the River Nith in New Cumnock, UK. The scour sensing system is capable of providing a continuous and direct measurement of the scour development at a bridge foundation under extreme events, including the refill (deposited) process. To the authors’ knowledge, this is the first time that smart electromagnetic probes were employed in a real case-study.

## 2. Scour Monitoring Techniques

**Figure 1:**
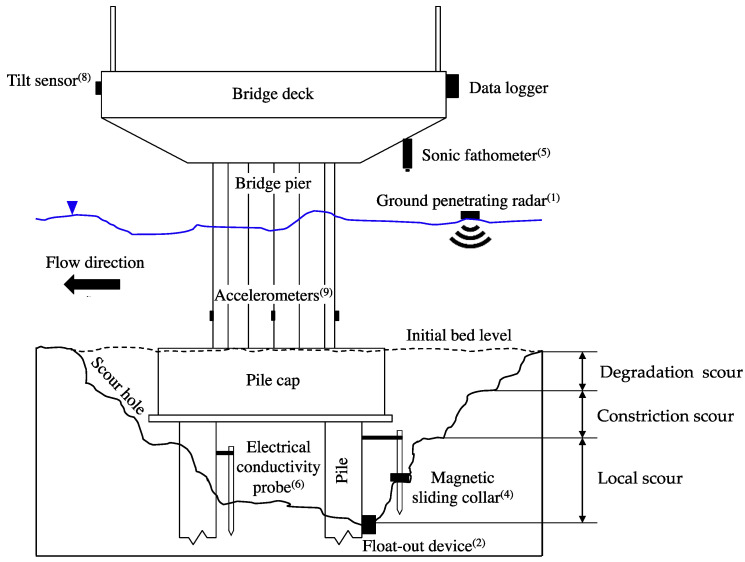
Schematic illustration of type of scour and bridge scour monitoring devices.

A wide range of techniques have been developed in the last few decades for monitoring bridge scour (see e.g., Prendergast and Gavin [15] for a state-of-the-art review). Many of these techniques provide a direct measurement of the scour depth at a bridge pier, whereas other techniques provide information on the effects of scour on the bridge. Table 1 reports the most widespread techniques with relevant references, and Figure 1 illustrates a bridge pier equipped with some of these sensors.

To be effective, bridge scour monitoring should provide continuous real-time data with a good resolution, especially during a peak flood event. Detecting the presence of redeposited soil can also deliver beneficial information about the foundation bearing capacity. Table 1 reviews the scour monitoring techniques based on the features that quantify their reliability and define their field of application. Among those, the table outlines the ability of the devices to provide a continuous monitoring, their usefulness in identifying and monitoring the scour depth development during high flows as well as the capability to track the refill (deposition) process. Furthermore, the scour measurement resolution of each sensor is highlighted, where “High” defines a resolution better than 10 cm whereas “Low” means “order of tens of cm”. This property is not quantifiable for the indirect scour monitoring devices because they only detect change in the structural response (e.g., pier inclination or changes in bridge’s modal properties due to a certain level of scour), and typically recognised the presence of scour when it is so critical to affect the structural stability. The last column provides an indication of costs for the deployment of the monitoring technique (i.e., including installation costs).

**Figure 2:**
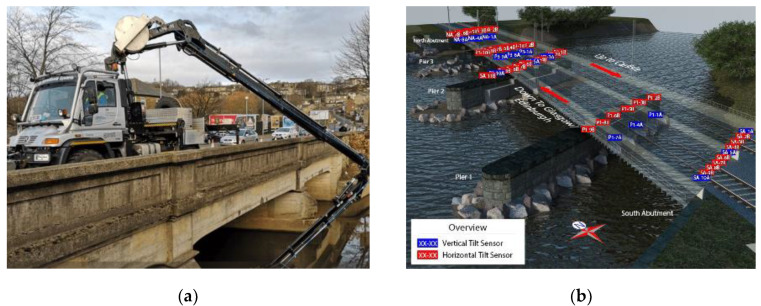
(**a**) BridgeCat technology for bridge inspection [40]; (**b**) Layout of the Structural Health Monitoring (SHM) system installed at the Lamington viaduct (courtesy of Network Rail).

Despite the development of such a wide range of sensors, practical applications aiming to monitor real-time bridge scour are very limited because of accessibility issues under flood events, damage, their cost and their inherent imprecision. However, there are few examples of use of scour monitoring systems, particularly in the UK, a country whose bridges have been affected significantly by scour in recent years [36,37] and where the use of scour sensors is increasing fast. Among these, it is worth mentioning the “BridgeCat technology” [38], consisting of a vehicle featuring a hydraulic arm equipped with a mechanical scanning sonar, a high-resolution camera, and a digital altimeter measuring height off the riverbed (Figure 2a), the network of tilt sensors for detecting structure movement caused by scour in Lamington Viaduct [14] (Figure 2b), and the vibration-based scour detection system deployed for five months at the Baildon bridge in Bradford, UK [39].

In summary, few technologies are able to make a scour detection with a resolution better than 10 cm while at the same time are able to separate the redeposited soil and saturated soil. Among these, the dielectric probes are the only ones which allow for recording during an extreme event and thus can be used for an early warning system. Although very appealing to date, this class of sensors has only been tested in the laboratory. Hence, in this paper we present the application of these dielectric probes to a real world setting and we critically discuss its performance.

## 3. Pilot Scour Monitoring System

The sensing system described in this article is based on the use of a dielectric probe equipped with capacitive sensors, which represent one of the techniques available for measuring electromagnetic properties of the soil [41]. The term “capacitive” refers to the working principle of the electric device, which can be exemplified by considering an LC circuit (L = Inductor, C = Capacitor) [41]. The resonant frequency of the LC circuit depends on the dielectric permittivity of the medium interposed between the two capacitor conductors.

**Figure 3:**
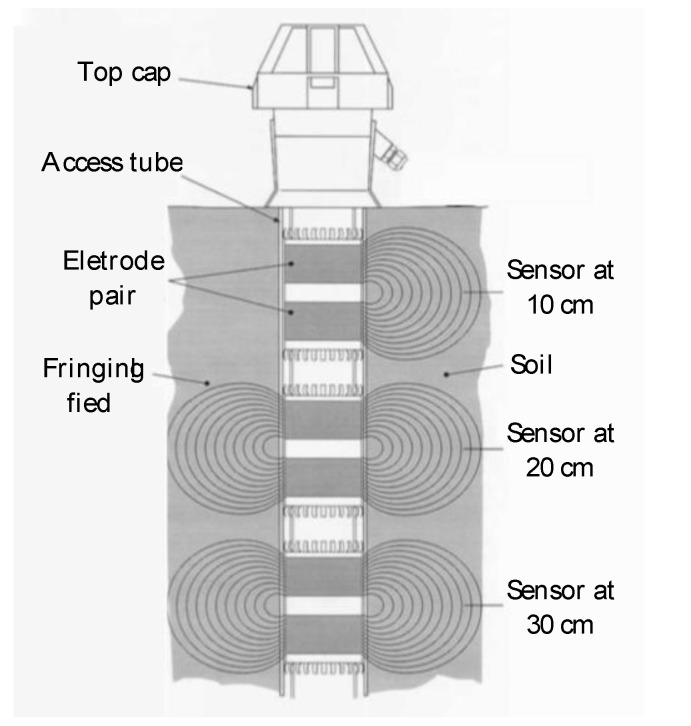
Schematic of the dielectric probe equipped with electromagnetic sensors.

Each sensor is formed by an electrode pair (i.e., the two capacitor ring conductors) which transmits an electromagnetic fringing field that penetrates the external surrounding medium (see Figure 3). Since the two electrode rings have diameter greater than their spacing, the capacitance is not only affected by the medium directly between the conductors (as is the case of the infinite conductors) but also on the medium surrounding the electrodes laterally. Since the configuration and geometry of the probe remain constant, any change in capacitance only depends on the dielectric property of the surrounding soil. The capacitor made of the two ring conductors is inserted into an LC-type circuit. The capacitance and, hence, the dielectric permittivity of the surrounding soil, is measured by the resonant frequency of the circuit via an oscillator inserted into the LC circuit as discussed by Tarantino et al. [42]. The term “electromagnetic sensor” is used hereinafter to refer to these smart probes, since they are used in this context to measure the dielectric permittivity (i.e., a soil electromagnetic property) to detect a scour hole.

The dielectric permittivity can therefore be measured if a calibration function is established to convert the resonant frequency read by the sensors into a permittivity value, which differs between the soil in the riverbed and the water [28]. The system is calibrated to detect erosion and deposition of riverbed sediment in different soil types and under temperature that would commonly occur in a real case-study scenario [28]. It also allows for distinction between in situ and redeposited bed material, providing useful information about the load-bearing capacity of bridge foundations. It is noteworthy that, although smart sensing bars with electromagnetic devices have already been proposed and studied [28], to the authors’ knowledge, this is the first time they have been applied to a real case study for the continuous monitoring of scour at a bridge location.

**Figure 4:**
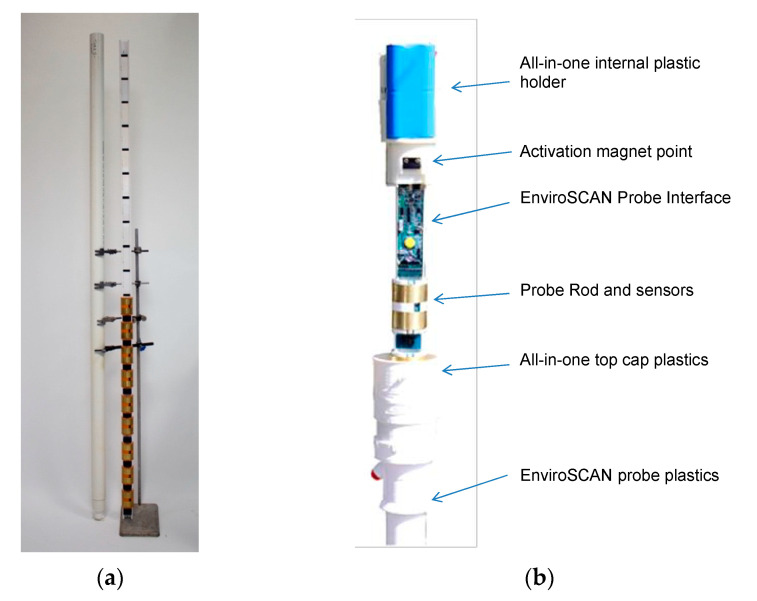
(**a**) The EnviroSCAN probe; (**b**) Probe’s components [44].

The electromagnetic device installed in the A76 200 bridge is the EnviroSCAN probe (Figure 4a), developed by Sentek sensor technologies [43] and provided by Soil Moisture Sense in the UK. The probe is different from the one used by Michalis et al. [28] since Soil Moisture Sense was the only supplier providing a bespoke version (i.e., customisable length) of the sensing bar. However, these two probes share a similar working principle. Every component of sensing bar is described in the following sections, including the experiments performed in the laboratory to calibrate each electromagnetic sensor and to test the smart probe’s functioning in a real-case scour scenario. The laboratory tests have been carried out on an improved version of the protocols used in Michalis et al. [28] to calibrate and test a similar capacitance probe.

The probe consists of a plastic rod equipped with multiple sensors, installed every 10 cm along the rod height. Therefore, the monitoring system has a resolution of 10 cm, but a smaller resolution (e.g., 5 cm) can be achieved when sensors read intermediate values of permittivity. The EnviroSCAN probe is supplied with a maximum of 16 sensors because its mainboard has 16 channels. However, the arrangement of the sensor is customisable since the plastic rod has several slots (at 10 cm to each other) where to insert a sensor. This feature makes the probe very versatile because different configurations can be achieved, such as a probe with 1.60-metre-long monitoring part with 10-cm resolution (i.e., 16 sensors installed without empty slots among them) or with a 3.20-metre-long monitoring part with 20-cm resolution (i.e., an empty slot after each sensor). Therefore, the more extended is the monitoring part, the lower the resolution of the system.

Figure 4b shows the components of the scour probe, which includes a battery, an electronic board (which is the EnviroSCAN Probe Interface), a 3G modem, and the electromagnetic sensors. The probe has an extended access tube made of plastic (i.e., its external diameter is equal to 56.5 mm) which protects the components of the probe (as shown in Figure 4a) from water damage and debris when it is installed in wet environments for monitoring purposes. The sensor is in the shape of a cylinder in order to fit closely inside this access tube.

**Figure 5:**
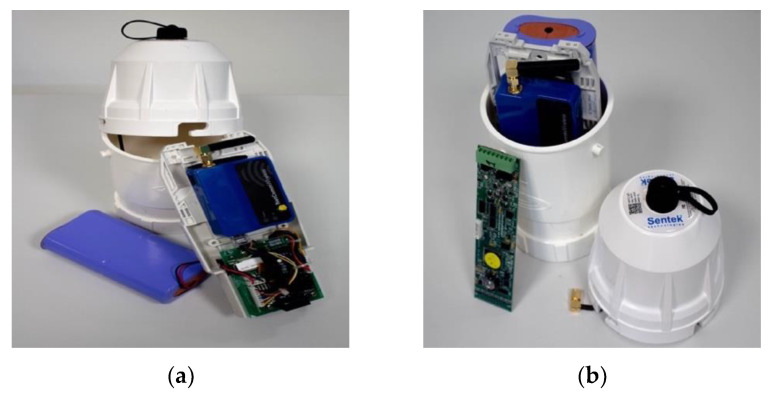
(**a**) The modem and battery; (**b**) The plastic holder and the electronic interface board.

Figure 5a shows the 3G modem along with an electronic board, the battery and the internal antenna, while Figure 5b shows the plastic holder protecting the battery and the EnviroSCAN Probe Interface electronic board. The components shown in Figure 5a comprise the Data Transmission Unit (DTU), called Sentek PLUS All-in-One. The sensor data can be stored in the probe, but they can also be sent to an ftp server thanks to the 3G modem that the DTU is equipped with. The probe uploads a .esp file at every reading, and then the data are converted in .csv/.xslx format through a dedicated software provided by the Soil Moisture Sense.

**Figure 6:**
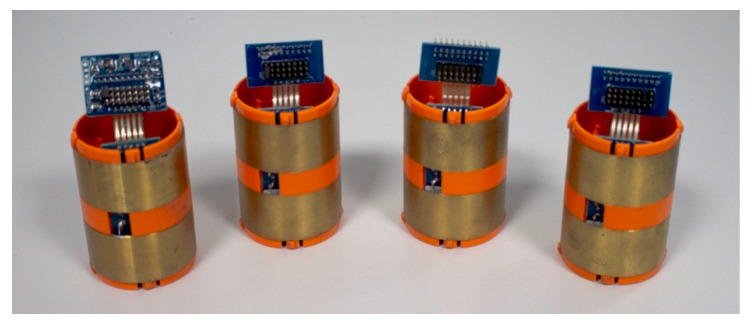
The electromagnetic sensors.

The DTU is also equipped with a Bluetooth module that is used for wireless testing and configuration, using a laptop with the Probe Configuration Utility (i.e., the Sentek probe configuration app). The data logger is also equipped with a high-capacity Lithium battery (14V 14 Ah), which provides energy supply to the probe and to the DTU for every data uploading. It generally lasts 12 months when using Sentek’s standard configuration (five sensors sampling every 30 min and upload interval of three hours) [44]. Figure 6 shows the electromagnetic sensors that measure the frequency of the surrounding medium.

### 3.1. Permittivity of Soil

The permittivity of a material (*ε*)*,* defined as its capability to polarise when exposed to an electrical field, is a dimensionless variable [45]. Average values of the (static) dielectric permittivity of water, solids, and air are respectively ε_w_ ≈ 78, ε_s_ ≈ 3 – 5, and ε_a_ ≈ 1. The bulk permittivity of soil *ε_s_* depends on the dielectric permittivity of its constituents (free water, bonded water, solids, and air) and their volume fraction (represented by the dry density, volumetric water content, and surface area). Since the dielectric permittivity of free water depends on temperature, ion concentration, and electromagnetic frequency, so does the bulk dielectric permittivity of the soil. However, the parameter that has the most substantial influence on *ε_s_* is the volumetric water content *θ*. It is worth noting that the term “permittivity” will hereinafter refer to the dimensionless “relative dielectric permittivity” *ε_r_*, which is the permittivity of a material relative to vacuum permittivity.

A three-phase model can be used to define the bulk permittivity (*ε_m_*) of a soil mixture having a negligible amount of bonded water (i.e., negligible fraction of active clay) [46]:

(1) $$\varepsilon_{m}^{\alpha }=\theta \times \varepsilon_{w}^{\alpha }+\left(1-\eta \right)\times \varepsilon_{s}^{\alpha }+\left(\eta –\theta \right)\times \varepsilon_{a}^{\alpha }$$

where:

- *θ* is the volumetric water content,
- *η* is the porosity,
- α is a dimensionless coefficient ranging from +1 to −1, which is positive when the electric field is perpendicular to the soil layer and negative when the electric field is parallel to the soil layer.

The volume fraction that corresponds to the solid phase is (1 - *η*), whereas (*η* - *θ*) is the volume fraction that corresponds to the air phase. When the soil is saturated (*η* = *θ*), Equation (1) reduces to the following one:

(2) $$\varepsilon_{m}^{\alpha }=\eta \times \varepsilon_{w}^{\alpha }+\left(1-\eta \right)\times \varepsilon_{s}^{\alpha }$$

It is instructive to calculate the soil bulk dielectric permittivity using Equation (2) for the cases where the electromagnetic sensor is surrounded by (i) in situ soil sediment (pre-scour), (ii) water (soil washed away due to scour), and (iii) redeposited soil sediment having higher porosity (post-scour). These are given in Table 2 assuming α = 0.50, *ε_s_* = 4, and *ε_w_* ≈ 78 and porosities in the range η = 0.4–0.5 for the original riverbed soil before scouring and η = 0.5–0.6 for the redeposited sediment.

**Figure 7:**
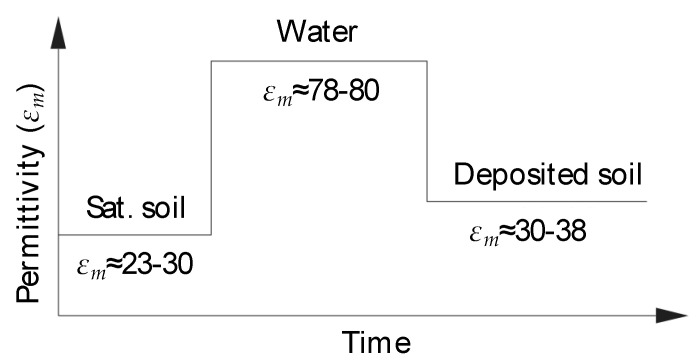
Variation of permittivity over time during scouring action.

The significant dissimilarity in bulk permittivity before, upon, and post scouring is used to detect scour and bed material deposition processes surrounding the foundations. An example of the time history of the permittivity values before, during and after a scouring process is shown in Figure 7. It can also be observed that the pre-scour and post-scour conditions correspond to quite different permittivity values, which is useful to identify whether the scour hole has been refilled.

### 3.2. Scaled Frequency N

As mentioned before, the electromagnetic sensor provides information on the permittivity of the medium around it by measuring the resonant frequency. The EnviroSCAN probe retunes the resonant frequency of the surrounding medium in terms of scaled frequency that depends on the frequency of water and air [47]. The scaled frequency *N^k^* (for the *k* = 1,2,…,*N* sensor) is evaluated according to the expression below:

(3) $$N^{k}=\frac{R_{A}^{k}-R_{E}^{k}}{R_{A}^{k}-R_{W}^{k}}$$

where:

- *R^k^_A_* is the resonant frequency of air read by the sensor *k*,
- *R^k^_W_* is the resonant frequency of water read by the sensor *k*,
- *R^k^_E_* is the resonant frequency of the field read by the sensor *k*.

*N^k^* is a dimensionless number that varies depending on the dielectric permittivity of the medium surrounding the sensors (with *N^k^* = 0 when the sensor is in air and *N^k^* = 1 when it is submerged in pure water).

### 3.3. Calibration of Scour Probe

A calibration is required to establish the correlation between the bulk permittivity of the soil mixture (*ε_m_*) to the scaled frequency (*N^k^*) collected by the sensors of the EnviroSCAN probe. The calibration study was conducted in the geomechanical laboratory located at the University of Strathclyde, Glasgow, prior to installation in the bridge site, by exposing the probe to various media using different chemicals of known dielectric permittivity and by measuring the corresponding sensor readings [28]. The scaled frequency *N^k^* corresponding to each chemical is calculated with Equation (3), where the values of *R^k^_A_* and *R^k^_W_* have been recorded in the laboratory during the calibration phase by submerging the probe in fresh water and air. The chemicals used for the experiment are Acetone, Acetonitrile and Methanol. The permittivity values of these abovementioned chemicals are shown in Table 3, together with those of water and air.

The scaled frequency readings of the probe were plotted in a graph against the values of the permittivity of the considered chemicals and the following analytical relationship was fitted [41], consisting of a quadratic and an exponential factor:

(4) $$\varepsilon_{m}\left(N\right)=\left(a_{0}+a_{1}\times N+a_{2}\times N^{2}\right)\;\times e^{kN}$$

It is worth recalling that the scaled sensor reading has two extreme values, i.e., *N* = 0 when the sensor is in air, and *N* = 1 when it is in pure water. Thus, in accordance with Equation (3), Equation (4) must fulfil two constraints: *ε_air_*(*N* = 0) = 1 and *ε_water_*(*N* = 1) = 78.4. Therefore, the values of the parameters *a*_0_ and *a*_1_ can be evaluated and Equation (4) can be reduced to:

(5) $$\varepsilon_{m}\left(N\right)=\left(\varepsilon_{air}+\left(\varepsilon_{water}\times e^{-k}-1-a_{2}\right)\times N+a_{2}\times N^{2}\right)\;\times e^{kN}$$

**Figure 8:**
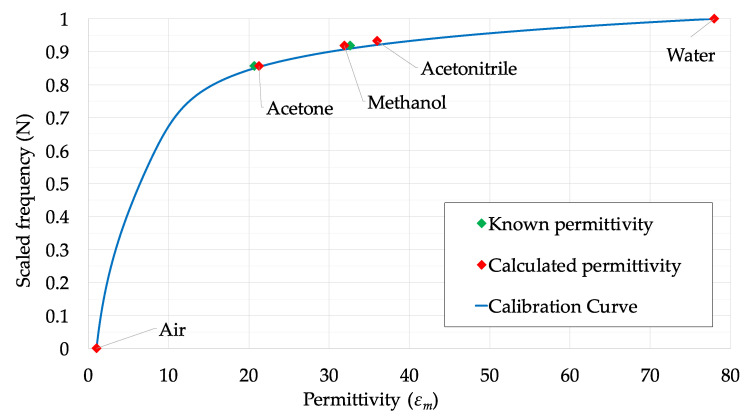
Calibration curve from calibrations tests.

Figure 8 shows the calibration curve obtained after conducting the experiment using the chemicals shown in Table 3. The green dots denote the values of the known permittivity of the chemicals plotted against the measured scaled sensor readings (*N*). Plotted in the same figure is the curve corresponding to Equation (5), with the parameters *a*_2_ = 1.2794 and *k* = 6.6537 fitted using the least square method. The values of the permittivity estimated by the proposed equations are very close to the known permittivity of the chemicals, as also shown in Table 4. Thus, Equation (5) can be used to convert the infield scaled sensor readings *N^k^* into values of permittivity using the fitted values of the parameters *a*_2_ and *k*.

### 3.4. Static Scour Test

**Figure 9:**
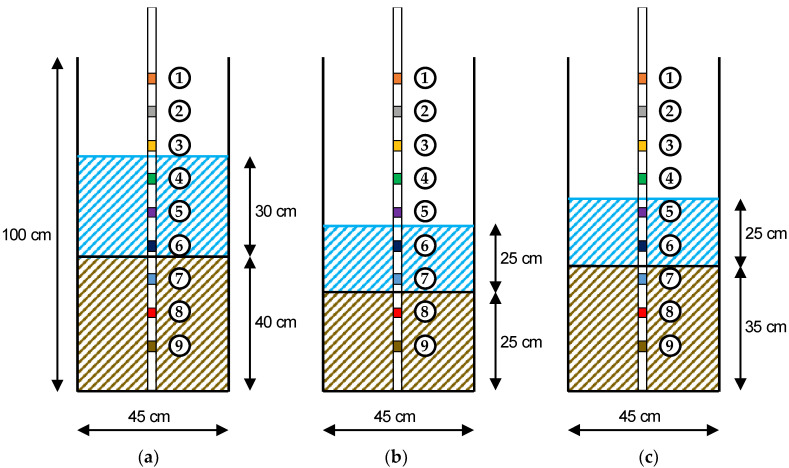
Experimental setup (**a**) the pre-scour, (**b**) the scour, and (**c**) the deposition condition.

A “static” scour test was performed in order to mimic probe functioning in a real-case scour scenario. Figure 9 shows the test setup used to record the probe response during a simulated scour event and the following deposition phase. The probe, equipped with nine electromagnetic sensors, was placed in the middle of a custom made cylindrical acrylic tank with diameter and height of 45 cm and 100 cm respectively. The initial setup consisted of three sensors in each medium, i.e., soil, water and air (Figure 9). The soil used for the experiment was silica sand, with a particle size of 1 mm.

The static scour test consisted of the removal of soil by hand in order to reproduce the scour process and, after recording the sensor response, of the manual refilling of the tank to simulate the deposition phase. The test protocol expanded on the one used by Michalis et al. [28] by highlighting the capability of the sensor to monitor the scour hole refill process (i.e., step 6). It was articulated in the following steps:

1. The probe was placed in the centre of the acrylic tank and kept vertical with the help of supports.
2. The acrylic tank was filled with silica sand for 40 cm. Each time a 10-cm layer of sand was added, it was compacted using a proctor hammer and a square-shaped plywood piece to ensure even compaction.
3. The soil was saturated with fresh water, and the tank was filled with 30 cm of water above the sand surface to simulate a static soil–water interface, such as the riverbed condition (Figure 9a).
4. The probe recorded the sensors’ response for ten minutes (i.e., “pre-scour condition”).
5. The scour process was started by manually removing a 15 cm layer of soil around the probe until a depth of about 25 cm was reached (Figure 9b). For this purpose, a small shovel was used. The “scour condition” lasted 20 min.
6. The deposition process was mimicked by partly refilling the layer of removed soil around the probe, reaching a total soil depth of 35 cm (Figure 9c). The response of sensors was recorded for ten minutes (i.e., “deposition condition”).

**Figure 10:**
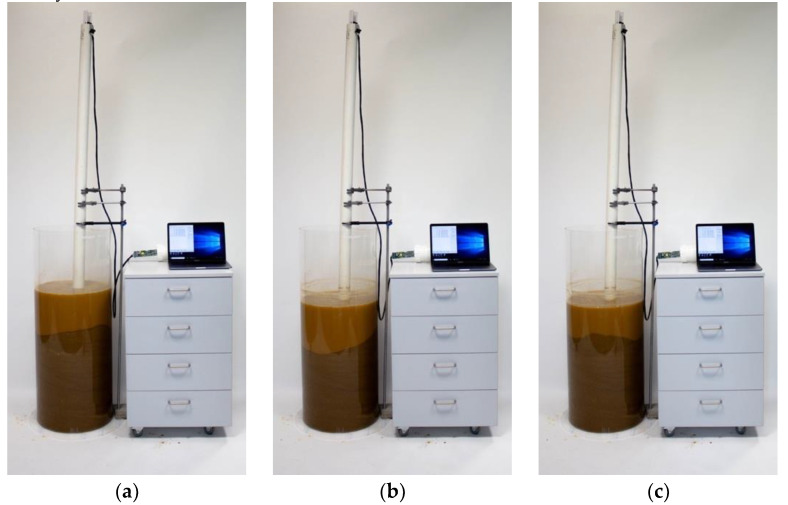
Static scour test showing (**a**) the pre-scour, (**b**) the scour, and (**c**) the deposition condition.

Figure 10 shows the three conditions achieved during the test, e.g., pre-scour, scour and deposition period.

**Figure 11:**
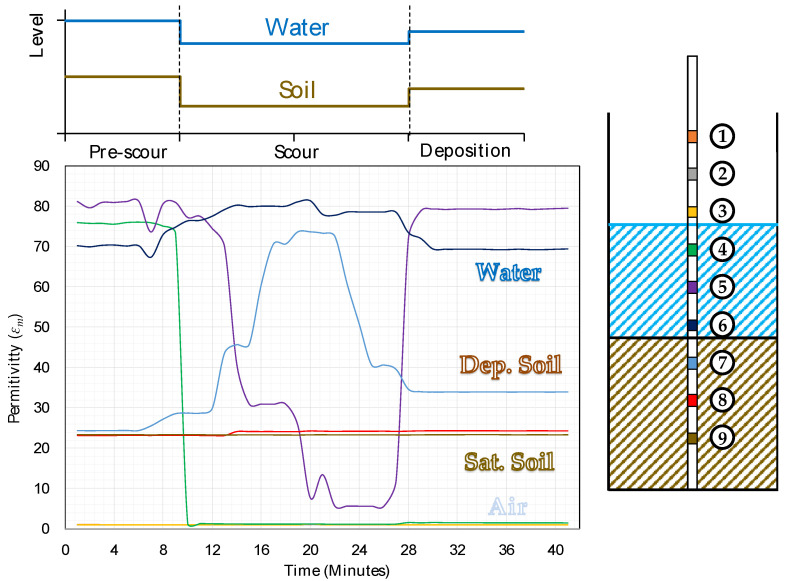
Permittivity *ε_m_* recorded by the nine sensors during the static scour test.

Figure 11 illustrates the time history of the permittivity values recorded during the test by the nine sensors in the probe. The values of permittivity shown in Figure 7 allows for identifying four bands in the plot, separating the sensor reading associated to the permittivity of water (*ε_m_* = 70–80), saturated soil (*ε_m_* = 23–30), deposited soil (*ε_m_* = 30–38) and air (*ε_m_* = 1). The scouring and deposition process did not affect sensors 1, 2 and 3 because they were in air for the duration of the whole test. This is confirmed by the value of the permittivity near one maintained throughout the experiment. Similarly, sensors 8 and 9 recorded a constant value of permittivity of approximately 23, i.e., the value of *ε_m_* associated with saturated soil.

The response of sensor 4 shows a drop in the permittivity from 75 (i.e., water) to 1 (i.e., air), that can be explained by the fact that the water level dropped after the soil was removed (see Figure 10b,c and the top graph of Figure 11). The initial value was not recovered after the scour hole was refilled by redepositing sand. The recording of sensor 5 exhibits a similar behaviour: it starts in water, it is in air during the scour condition because the water level falls but, when the soil is refilled, the water level slightly rises, and the sensor is again submerged in water.

Sensor 6 stayed in water for the whole test duration, and the recording of its permittivity is almost constant. Some noise and signal disturbances can be noted during the scour condition due to the manual excavation and refilling (i.e., the mixture of water, soil and air bubbles might explain the fluctuations of the signal).

Sensor 7 is the sensor where the three different conditions simulated in the test can be observed. Initially, during the pre-scour period, the permittivity is around 23 (i.e., within the permittivity range of saturated soils). When the scour is simulated, the signal record starts increasing and reaches a value of 74, which falls in the water range. During the excavation actions, and when the soil is later filled as well, the sensor registers intermediate values of permittivity because when the soil is removed or repositioned, the surrounding medium is a mixture of water and soil. Finally, during the deposition period, the permittivity decreases to a value in the range of deposited soil (i.e., *ε_m_* = 30–38).

## 4. A76 200 Bridge

**Figure 12:**
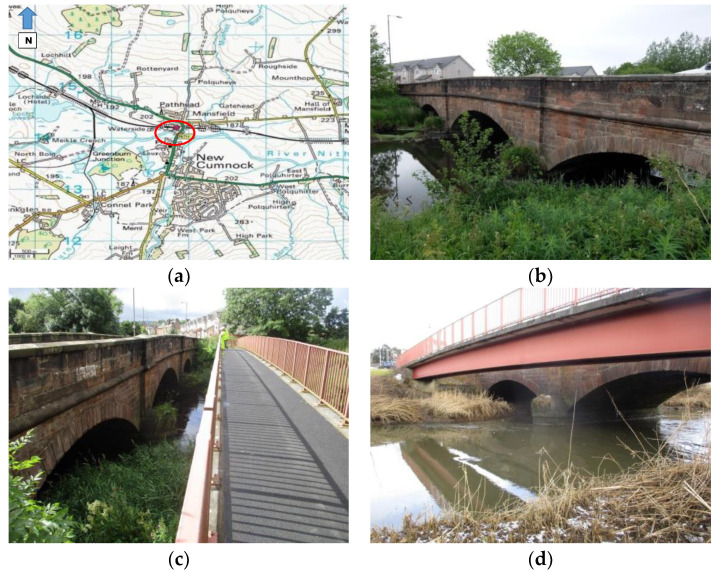
(**a**) Location of the A76 200 Bridge; (**b**) Side view of the A76 200 Bridge; (**c**) The pedestrian bridge; (**d**) Side view of the two bridges.

The A76 200 bridge is a 3-span stone masonry arch carrying the A76 two-lane single carriageway over River Nith in the small village of New Cumnock in the South-West of Scotland (Figure 12a). The middle span is 10.70m long, whereas each of the approaching spans is 9.10m long. The span width is 8.5m between the outer faces of the spandrel walls. The arches are formed of ashlar stonework with punched face and chamfered edges, as shown in Figure 12b. Abutments and piers are all founded on spread footings on the natural riverbed and based on previous inspections that have experienced significant scour in the past.

A total of 3.5 metres upstream of the A76 200 bridge (Figure 12c) there is a pedestrian bridge. This is a single span, simply supported composite structure, with a clear span of 34 m. The deck consists of precast concrete deck units supported on two I-beams at 1250 mm centre-to-centre distance. The beams have a rectangular section, 920 mm high and 350 mm wide. The precast concrete deck units are formed with a slight up-stands, on which the pedestrian parapets are bolted (Figure 12d).

**Figure 13:**
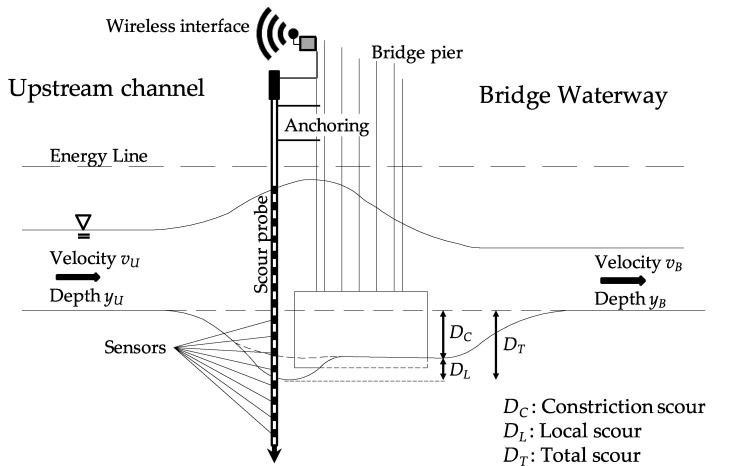
Outline of the pilot scour monitoring system.

The scour monitoring system consists of two four-metre-long scour probes that are equipped with electromagnetic sensors installed along the plastic rod height. Therefore, there are sensors buried into the riverbed and others within or above the running water of the River Nith. The former sensors can detect the scour depth, whereas the latter ones, being able to discriminate the permittivity values between air and water, can be used to measure the water level (Figure 13). The smart probe is protected from water by a plastic tube. The probe and the tube are encased in a circular hollow section metal tube, which is well secured at the top to the piers to prevent any movement and to ensure stability during a flood event.

**Figure 14:**
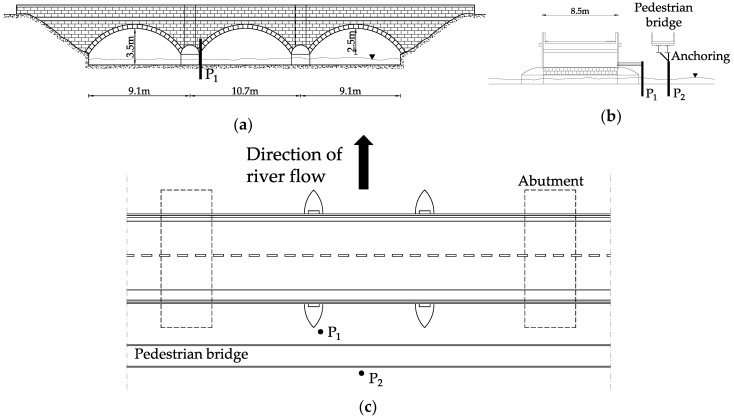
Layout of the pilot scour monitoring system. (**a**) Side view; (**b**) Section view; (**c**) Plan view.

**Figure 15:**
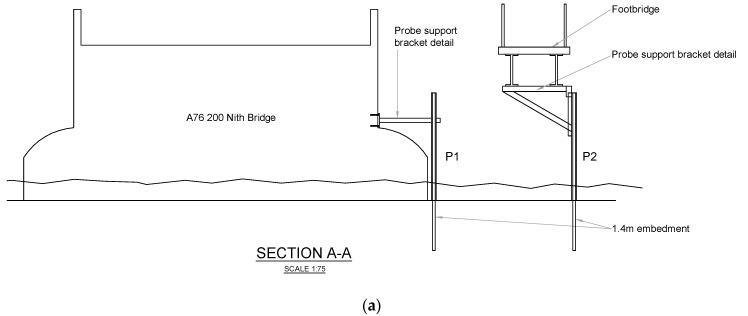

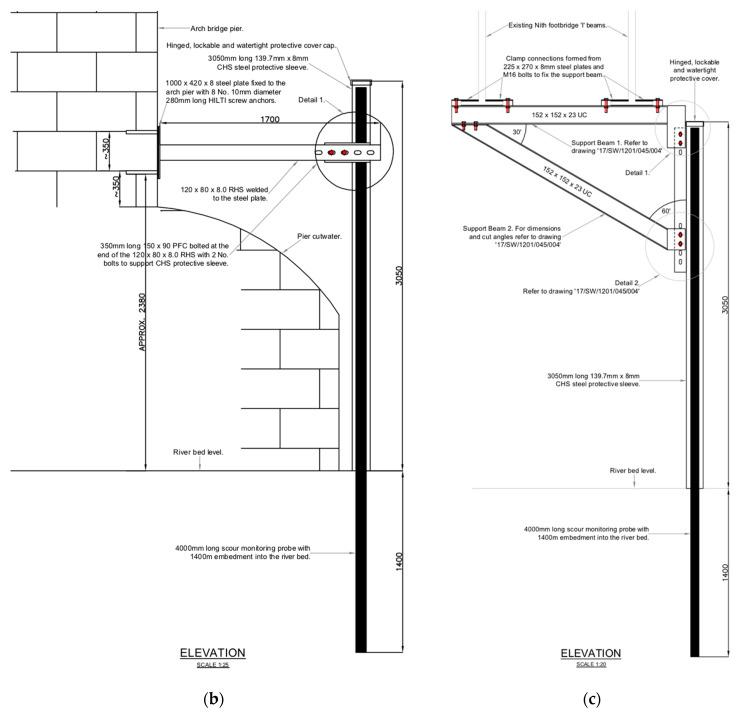
(**a**) Anchorage system for the probes; (**b**) Elevation of anchorage system for probe P1; (**c**) Elevation of anchorage system for probe P2.

One probe (P1) is installed on the upstream face of a pier of the A76 200 bridge to detect total scour, whereas the other (P2) is installed in the centre of the river to detect degradation and contraction scour, and is connected to the pedestrian bridge (Figure 14). Figure 15 shows the details of the connections between the two probes and the bridges, ensuring their stability during a flood event.

Probe P1 is equipped with 11 sensors (four sensors buried into the riverbed), whereas probe P2 with 15 sensors (three sensors into the riverbed). Probe 1 is able to monitor up to 40 cm of scour depth, whereas Probe 2 can detect a maximum scour depth of 30 cm.

**Figure 16:**
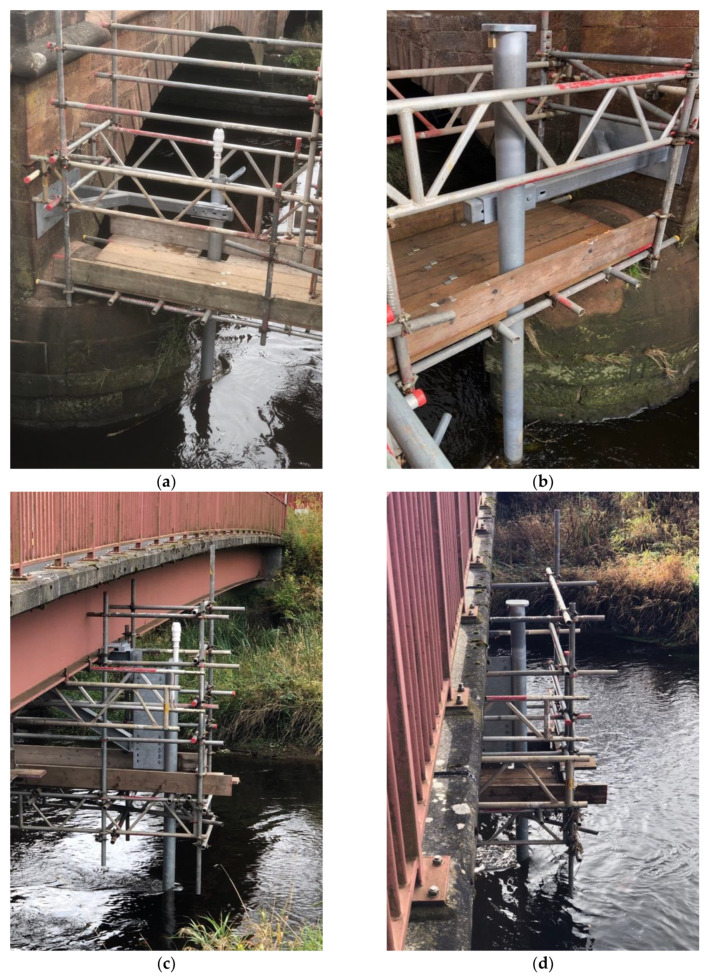
(**a**) and (**b**) Probe P1; (**c**) and (**d**) Probe P2.

The two probes were installed on the 4th of October 2018, and the monitoring of the scour depth started on the same day (Figure 16). In January 2019, the probes were equipped with an external antenna to improve the 3G modem signal for the uploading of the data. The battery of probe P1 reached its end of life at the beginning of November, thus exceeding the expected lifespan according to the DTU’s user manual [44]. Close to the end of its life, data were still recorded, but the battery voltage was not enough to transmit to the cloud. As a result, data from 7th November 2019 to 11th December 2019 were lost because the memory in the probe’s motherboard has limited space (i.e., 2000 readings) and new readings overwrite the oldest reading first when the storage is full.

## 5. Scour Data Analysis

### 5.1. Probe 2 in the Middle of the Channel

**Figure 17:**
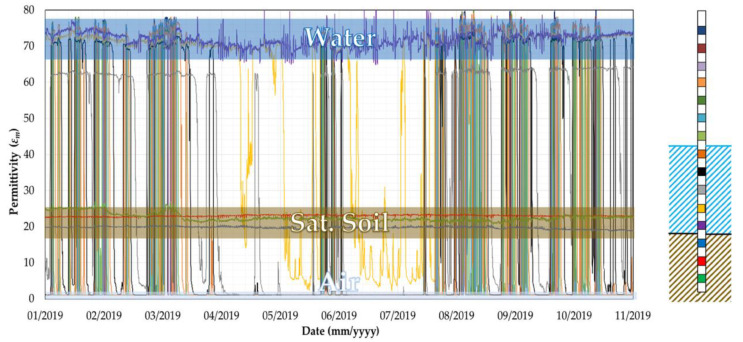
Readings of the 15 sensors installed in Probe 2.

Figure 17 shows the sensors readings collected by probe P2. According to the different values of permittivity shown in Figure 7, three bands can be identified, associated with the permittivity of water (*ε_m_* = 70–80), saturated soil (*ε_m_* = 20–27) and air (*ε_m_* = 1).

**Figure 18:**
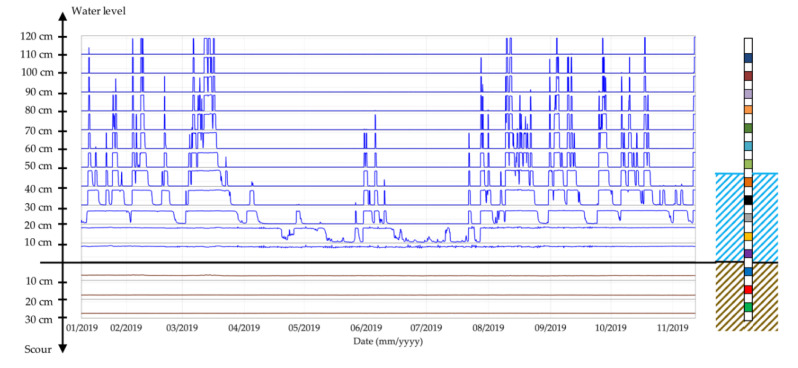
Rise and fall of water detect by the sensors of Probe 2.

Thanks to the sensor capacity of reading the permittivity of water and air, it is possible to discriminate the status of a sensor between these two environments and, essentially, the probe can also be used as a water level detector, as shown in Figure 18. The only drawback of this monitoring technique is the limited number of sensors involved for water level measurements; the main board of the probe has 16 channels, which poses a limit to the number of sensors that can be deployed. Considering that some of them are buried in the riverbed for detecting the erosion, only a few electromagnetic sensors can be employed to measure the rise and fall of river water. For instance, Probe 2 can measure a maximum water level equal to 120 cm.

**Figure 19:**
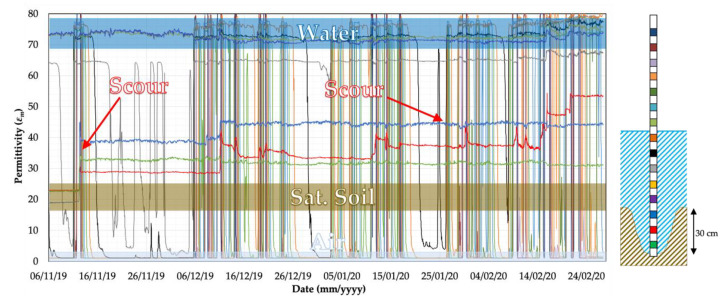
Detection of scour hole by Probe 2.

On the 12th of November 2019 at 4 am, the three sensors of probe P2 located into the riverbed registered a change in the value of permittivity. The jump of the three sensor readings (green, red and blue signal in Figure 19) indicates the presence of a scour hole of 30 cm (i.e., the spacing among sensors is 10 cm).

### 5.2. Probe 1 at the Bridge Pier

**Figure 20:**
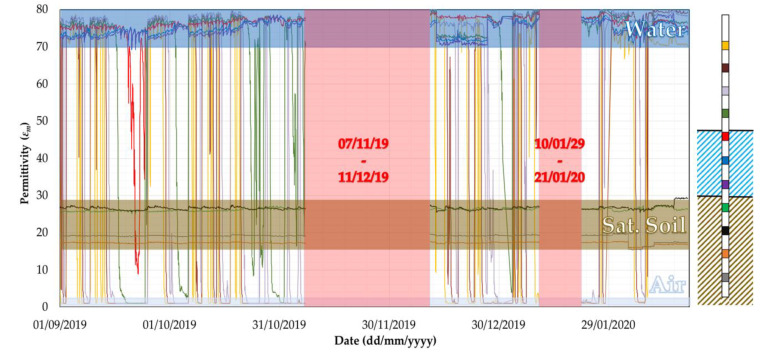
Readings of the 11 sensors installed into Probe 1.

The probe P1 installed at the pier of the A76 200 Bridge has not recorded any scour. As observed in Figure 20, the signals of the last four sensors have never changed from the value of permittivity for saturated soil (ε = 20–27). Unfortunately, the battery of Probe 1 reached its end of life a few days before the peak flooding event (12th of November) and the scour data were stored on the probe’s motherboard. However, the maximum capacity of the internal memory, able to store two months of data, was reached, and this explains why around one month of data were lost. Once the battery was replaced most of the data were recovered, and it can be seen that the permittivity values are still very close to those recorded in November before the failure of the battery.

## 6. Discussion

**Figure 21:**
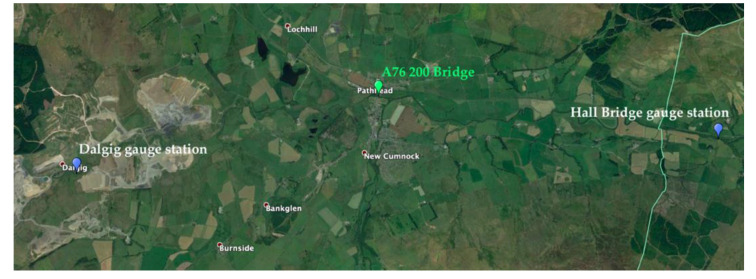
Map of Scottish Environmental Protection Agency (SEPA) gauging station.

**Figure 22:**
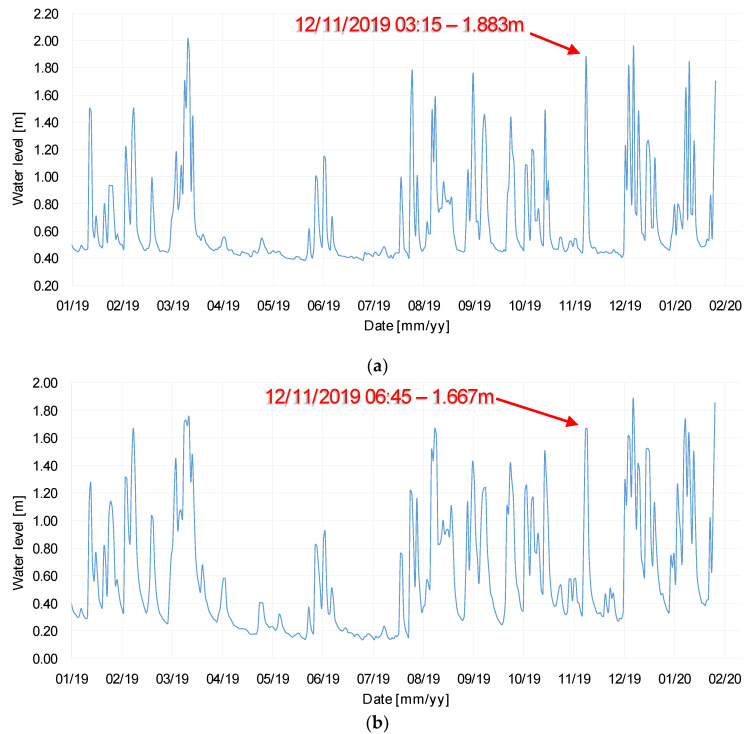
(**a**) Water level time-history at Dalgig station; (**b**) and Hall Bridge station from Jan. 2019.

Data concerning the maximum daily value of river stage provided by the Scottish Environmental Protection Agency (SEPA) show a peak in the water level of the river during the night before the 12th November, which could have caused the scour event registered by probe P2. In particular, the gauging station located in Dalgig (upstream to the A76 200 bridge, see Figure 21) registered a value of water level equal to 1.883 m at the 3:15 am of the 12th November 2019, as shown in Figure 22a. The gauging station located in Hall Bridge (downstream to the A76 200 bridge, see Figure 21) registered a peak value of water level equal to 1.667 m at the 6:45 am of the 12th November 2019, as shown in Figure 22b. Both values are the maximum values registered by the gauging stations since March 2019, as can be seen from the graph in Figure 22a,b. Furthermore, the water levels recorded by the two gauging stations have been exceeded only ten times at Dalgig and 16 times at Hall bridge in the last six years.

### Inspection and Visual Check at the Bridge Site

**Figure 23:**
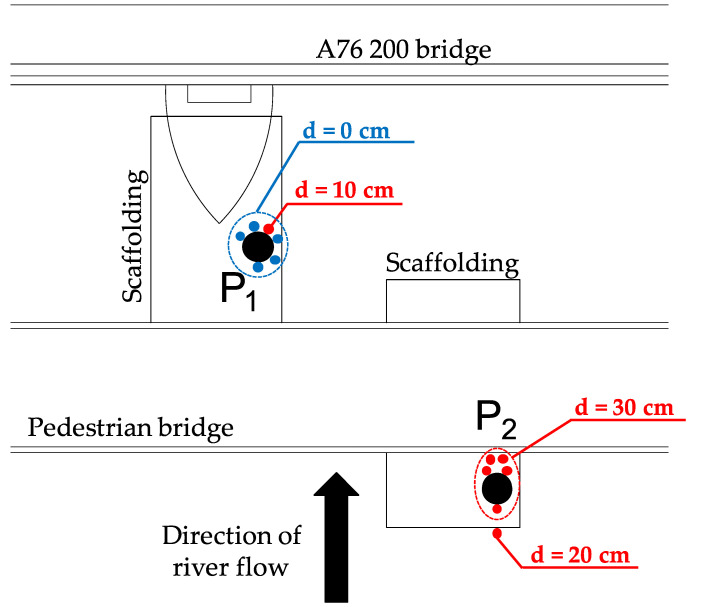
Locations and scour measurements carried out during the probe inspection.

In order to verify the recorded data and check the scour recorded by the two probes, the A76 200 bridge was visually inspected and the riverbed depth was measured at the two probe locations. A 3.6-metre-long telescopic pole was used to evaluate the scour at the locations illustrated in Figure 23, based on the comparison between the initial river bed depth at the time of probe installation and at the time of the new measurement. The measured values of the scour are reported in Table 5.

The data obtained by the probe P2 are supported by the actual measurement of scour in the vicinity of the steel tube and immediately downstream to it. Five measurements provided the same value of scour depth (i.e., 30 cm). A measurement was taken upstream from the tube, just over the scaffolding, and 20 cm of scour was recorded. The recorded scour might be the result of the turbulence of water around the steel pipe, since a circular tube inserted into the riverbed can induce local scour depths up to 2.5 times its diameter [48]. The diameter of the tube is 139 mm, which therefore may lead to a scour depth of 35 cm. However, this pipe-induced scour does not invalidate the obtained results, and in fact, it has confirmed the probe’s capacity to detect scour.

**Figure 24:**
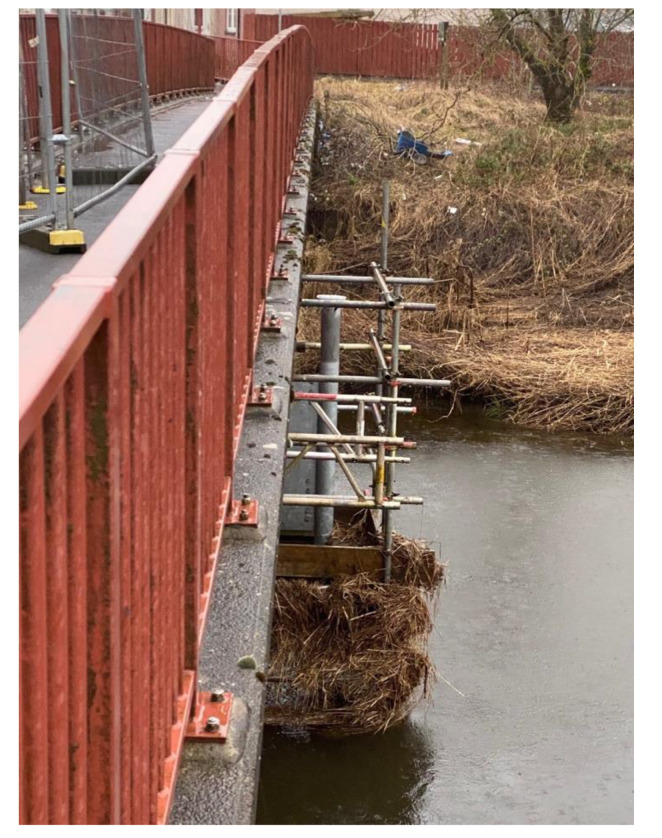
Hay and debris on the Probe 2.

Moreover, the abundant presence of debris and particularly of hay on the scaffolding and the steel tube (see Figure 24) may explain the recorded values of the permittivity of the last three sensors of P2, which were around 50 (Figure 19) and did not reach the water permittivity reference value (*ε_w_* = 70–80). Nevertheless, this value is higher than the permittivity of the saturated soil, allowing the probe to detect the scour.

Finally, a small and highly localised scour hole (i.e., less than 5 cm of diameter) was found on the downstream side of the steel tube protecting probe P1. This may be due to the local erosion induced by the turbulence of water around the steel pipe as well. However, the remaining soil surrounding the tube was not found to be scoured, and this explains why the small hole did not influence the readings of probe P1.

It is worth mentioning that using the formula of BD97/12 [49], a total pier scour depth equal to 2.93 m is obtained under the discharge corresponding to the peak flood event registered in the early morning of 12th November. This outcome shows that empirical formulas embedded in current assessment procedures may result in overconservative scour estimates. This is mainly because they are based on laboratory tests under controlled conditions that are not representative of real ones (see e.g., [4]). Thus, the information from scour monitoring at bridge foundations could be very useful to reduce the bias and improve scour estimation models [50].

## 7. Conclusions

Few monitoring technologies can detect scour with a good resolution (e.g., better than 10 cm) and also discriminate between the saturated and redeposited soil. Among these, only the dielectric probes allow recording during an extreme event and thus can be used for an early warning system. Although very appealing, this class of sensors was not tested in a real world setting before this study.

This paper presents the concept, functioning and output of a pilot scour monitoring system based on dielectric probes and installed on the A76 200 bridge over the River Nith in New Cumnock, UK. This monitoring technique tracks the evolution of the scour depth by detecting changes in the medium permittivity surrounding bridge foundations and can also distinguish between air, water, saturated soil and redeposited soil, which is useful to assess whether the scour hole has been refilled after the flood peak has receded.

After a brief review of the main features of the most diffused scour monitoring techniques, the paper introduces the principle of operation of the dielectric probes, together with the procedure implemented for calibrating and testing the sensors. A “static” scour test is carried out to evaluate the capability of the sensor to monitor the scour hole development as well as the refill process, showing the potential of the probe in discriminating among air, water, saturated and redeposited soil.

The final part of the paper describes the pilot scour monitoring system installed in the A76 200 Bridge in New Cumnock (South-West Scotland). The system consists of two bespoke probes, one measuring the total scour at one pier (i.e., probe P1) and one monitoring the constriction scour in the middle of the channel (i.e., probe P2). After a peak flood event, the latter probe measured 30 cm of scour, and the recorded data are consistent with the actual measurement of scour in the vicinity of the probe carried out using a telescopic pole during a bridge inspection. This proves the potential of the technology in providing continuous scour monitoring, even during extreme flood events, thus avoiding the deployment of divers for underwater examination. Furthermore, it is noteworthy that the data collected by the scour monitoring system have shown that empirical formulas overestimate the scour depth. Thus, the proposed scour monitoring systems can also potentially be employed to quantify the scour model errors and develop more accurate scour estimation formulas.

Even though the recorded scour might be the result of the turbulence of water around the steel pipe, this pipe-induced scour does not invalidate the obtained results, and in fact, it confirms the probe capacity to detect scour. However, it raises a concern about the design of the protective system for the probe; an improved design must be pursued to have a system that protects the probe alone, and does not induce the scour to be monitored. Furthermore, the response of the sensors in the presence of very localised/nonuniform scour holes must be studied in a laboratory, as well as the influence of suspended sediments on the dielectric permittivity values.

The obtained data will be used to validate and improve current formulas for estimating the scour depth under transient flood conditions. Finally, these real-time measurements of scour depth will be used within a probabilistic framework for scour risk assessment to update, in real-time, the estimates of the scour depth at other locations of the bridge and other bridges.

## Figures and Tables

**Table 1:** Most widespread scour monitoring techniques.

	Continuousmonitoring	Measurement during extreme event	Scour depth resolution	Detectionof refill	Costs
**Direct Scour Measurement Devices**					
(1) Pulse or radar devices [16,17,18]	✔	✔	High		Medium
(2) Single-use or float-out devices [19]			Low		Medium
(3) Fiber-Bragg grating systems [20,21]	✔	✔	Low		Low
(4) Sounding or driven rod systems [19,22,23]	✔	✔	Medium		Medium
(5) Sound wave devices [24,25,26]	✔		High	✔	High
(6) Electrical conductivity devices [27]	✔		High	✔	Medium
(7) Dielectric probes [28]	✔	✔	High	✔	Medium
**Indirect Scour Measurement Devices**					
(8) Tilt sensors [19,24]	✔	✔			Low
(9) Accelerometers [26,29,30,31]	✔	✔			Low
(10) GPS [32,33]	✔	✔			Medium
(11) Satellite [34,35]	✔	✔			Low

**Table 2:** Values of calculated permittivity during scouring process.

Soil Condition	Porosity (*η*)	Permittivity (*ε_m_*)
Pre-Scouring (Saturated Soil)	0.40–0.50	23–30
Scouring (Soil is washed away)	1	78–80
Post-Scouring (Redeposited Soil)	0.50–0.60	30–38

**Table 3:** Known values of permittivity’s of different mediums.

Medium	Permittivity (*ε_m_*)
Air	1
Acetone	20.7
Methane	32.6
Acetonitrile	36
Water	78

**Table 4:** The values of known and calculated permittivity.

Medium	Scaled Sensor Readings (*N*)	Known Permittivity (*ε_m_*)	Calculated Permittivity (*ε_m_*)
Water	1	78	78
Acetonitrile	0.931	36	35.938
Methanol	0.918	32.6	31.863
Acetone	0.856	20.7	21.268
Air	0	1	1

**Table 5:** Scour inspection—Locations and scour measurements.

Location	Description	Scour Depth
P1	Highly localised hole around the steel tube	0–10 cm
P2	Large hole downstream the steel tube	30 cm
P2	Hole upstream the steel tube	20 cm
